# How to study biofilms: technological advancements in clinical biofilm research

**DOI:** 10.3389/fcimb.2023.1335389

**Published:** 2023-12-13

**Authors:** Leanne Cleaver, James A. Garnett

**Affiliations:** Centre for Host-Microbiome Interactions, Faculty of Dental, Oral & Craniofacial Sciences, King’s College London, London, United Kingdom

**Keywords:** biofilm, biofilm model, biofilm imaging, biofilm analysis, infection, host-microbe interactions

## Abstract

Biofilm formation is an important survival strategy commonly used by bacteria and fungi, which are embedded in a protective extracellular matrix of organic polymers. They are ubiquitous in nature, including humans and other animals, and they can be surface- and non-surface-associated, making them capable of growing in and on many different parts of the body. Biofilms are also complex, forming polymicrobial communities that are difficult to eradicate due to their unique growth dynamics, and clinical infections associated with biofilms are a huge burden in the healthcare setting, as they are often difficult to diagnose and to treat. Our understanding of biofilm formation and development is a fast-paced and important research focus. This review aims to describe the advancements in clinical biofilm research, including both *in vitro* and *in vivo* biofilm models, imaging techniques and techniques to analyse the biological functions of the biofilm.

## 1 Introduction

Biofilms are described as colonies of bacterial and fungal cells that adhere to and proliferate on both biological and non-biological materials (Costerton et al., 1995). Biofilms were first discovered centuries ago by Anton Von Leeuwenhoek, who used his microscope to examine tooth scrapings, but the first biofilm literature was not published until the 1970s, with the examination of “microbial films” taken from environmental water sources (Mack et al., 1975). However, the term “biofilm” was only introduced in the 1980s by Bill Costeron and collegues, which was used to describe microbial growth within medical samples (Lam et al., 1980; Marrie et al., 1982). More than 40 years on, we now realise that biofilms are the primary mode of growth for many bacterial and fungal species, which often form multikingdom communites with other organisms including protozoa and viruses (Burmølle et al., 2014). Therefore, understanding how biofilms form both inside and outide of the body has become an important aspect of modern medical research, such that new strategies can be developed to control environmental resevoirs of pathogens, and treat infectious diseases.

Biofilms go through a generalised life cycle. The classic model of biofilm development is a five-step process where biofilms resemble a mushroom-type shape. Planktonic cells (1) adhere, irreversibly, to surfaces which are primed with a conditioning film, followed by (2) proliferation of bacterial cells and an increase in biomass. This mass of cells then undergoes (3) maturation and (4) excretion of extracellular matrix. The final step is (5) dispersal of cells within the biofilm, allowing the dispersed planktonic cells the opportunity to adhere at other sites (Sauer et al., 2002; Stoodley et al., 2002). However, it is now thought that not all bacterial species grow in this classical way, especially within a native environment, and this concept has therefore been expanded on in recent years. A new conceptual model has been presented (Sauer et al., 2022), which instead suggests that there are 3 main stages of biofilm development; (1) aggregation and/or attachment, (2) growth and accumulation, and (3) disaggregation and/or detachment, with the possibility to aggregate and detach at any point and that these phases are affected by the environment and different conditions. This new model also accounts for non-surface-associated biofilms, particularly those observed in medicine, such as biofilms that form in the airways of patients with impaired mucociliary clearance (Sauer et al., 2022). Most importantly though, both models show that the maturation of biofilms, and the difference between simple aggregation and true biofilms, is defined by the excretion of extracellular polymeric substances (EPS), composed of carbohydrates, proteins, extracellular DNA (eDNA) and lipids (Di Martino, 2018).

Within the body a diverse microbial flora, arranged as biofilms, is associated with the skin and mucosal membranes, and these are highly beneficial to the host, for example within the gut (Macfarlane and Dillon, 2007), vagina (Leccese Terraf et al., 2016) and oral cavity (Robertson and McLean, 2015). However, dysbiosis can lead to changes within these biofilm communities, and can cause diseases such as inflammatory bowel disease (Baumgartner et al., 2021), bacterial vaginosis (Castro et al., 2019), and dental caries (Marsh, 2010), respectively. Bacteria can also cause a range of infections by forming biofilms on almost any indwelling medical device, including prosthetic joints, cardiac pacemakers, prosthetic heart valves, urinary catheters, and intravenous catheters (El Rafei et al., 2016; Beaver et al., 2021; Vilchez et al., 2021). These infections are often healthcare acquired (i.e. as a direct result of surgical contamination) but can also result from transient haematogenous spread of bacteria in the bloodstream (Honkanen et al., 2019). In addition, non-surface-associated bacterial aggregate biofilm infections are becoming more well-characterised (Alhede et al., 2011; Pabst et al., 2016; Cai, 2020), which are often chronic and low-grade, such as lung infections in cystic fibrosis patients (Høiby et al., 2010), chronic wound infections (Wei et al., 2019), and chronic otitis media (Akyıldız et al., 2013).

The type of infection being studied is important when choosing a biofilm model and screening technique, and some methods will be more appropriate than others. This review aims to discuss the different modelling techniques and the methods available for assessing functionality of biofilms.

## 2 Modelling biofilm growth

*In vitro* models for biofilm investigation can be highly tailored to the system that is being modelled. A multitude of variables can be chosen from, including different substrates on which biofilms can grow, mono- and poly-microbial seeding, seeding with bodily fluids, human-derived or artificial growth medium, chemically defined growth medium, and different culture conditions. Models can range from very simple systems to highly advanced biological simulations (**Table 1**).

**Table 1:** An overview of the analysis techniques included in this review, including the advantages and disadvantages of the different methods.

	Advantages	Disadvantages
Models		
Static (microtitre plates)	Cheap, easy, quick Batch culture Different substrates can be added and removed for imaging	Not true mature biofilms Limited nutrient availability
Dynamic	Flow cells – Constant nutrient flow, equipment is autoclavable, cheap and easy to set up. Bioreactors – Constant nutrient flow, additional biofilm analysis, ability to expose biofilms to different nutrients/antimicrobials etc. Microfluidics – Mimic *in vivo* biofilms *in vitro*, real-time imaging and growth dynamics, small inoculating and growth medium volumes, exposing biofilms to different nutrients/antimicrobials etc.	Flow cells – Contamination can be introduced easily. Bioreactors – Unacceptably large variation between biofilm of the same inoculum composition or inoculating sample type. Microfluidics – Risk of contamination (Subramanian et al., 2020) , can be significantly more expensive than basic models
Single species, mixed species and microcosm models	Single species biofilms optimise biofilm models, multispecies models mimic *in vivo*/infections, microcosm patient samples model infection directly from infection site.	Single species are not always the way bacteria grow naturally, chosen multispecies are representative and not complete microbiome.
*In vivo* modelling	More realistic and translational.	Moral and ethical issues with animal testing.
*Ex vivo* modelling	Explanted material more easy to work with, preservation of tissue structures, ability to detect host-responses.	Donor availability, deterioration of tissue samples, difficult to image biofilms deep in tissue samples (Grivel and Margolis, 2009) .
Imaging		
Fluorescence microscopy (CLSM and LLSM)	Easy and quick Increased resolution – single cell 3D imaging with CLSM and LLSM Ability to make as many fluorescent probes as possible Localisation of species/live/dead cells	Basic fluorescence has low resolution Overlapping fluorophores (photobleaching and phototoxicity) Intrinsic biofilm fluorescence
Electron microscopy	Highest resolution and magnification Intricate biofilm structural detail	Expensive Conventional SEM has long procedure times Destructive to sample Unable to analyse non-surface associated biofilms
Atomic force microscopy	High resolution Non-destructive Easy sample preparation	Adhered biofilm structures only Sample can become dried out
Scanning electrochemical microscopy	High resolution Non-destructive Metabolic potential	Adhered structures only Long scanning times Small scanning area
Genetic		
16S rRNA gene sequencing	Relatively cheap, quick, and easy to perform	Depending on sample, not always accurate identification to species level Primer/database bias
Metagenomic sequencing	All of the genes in a sample are identified	Not all genes are annotated depending on database used Not all genes present are transcribed
Transcriptomics and spatial transcriptomics	Gives a complete picture of gene expression Spatial transcriptomics shows where in the biofilm genes are up and down regulated	Expensive to run Bioinformatics is highly specialised Difficult, but not impossible, to achieve gene expression profiles down to single cells
Proteomics and Metabolomics		
Proteomics and spatial proteomics	Mass spectrometry is very sensitive There are many different mass spectrometry platforms available	Mass spectrometry destroys samples
NMR	Easy and reproducible to perform Retention of sample post-analysis	Not as sensitive as mass spectrometry

### 2.1 Static growth

**Figure 1:**
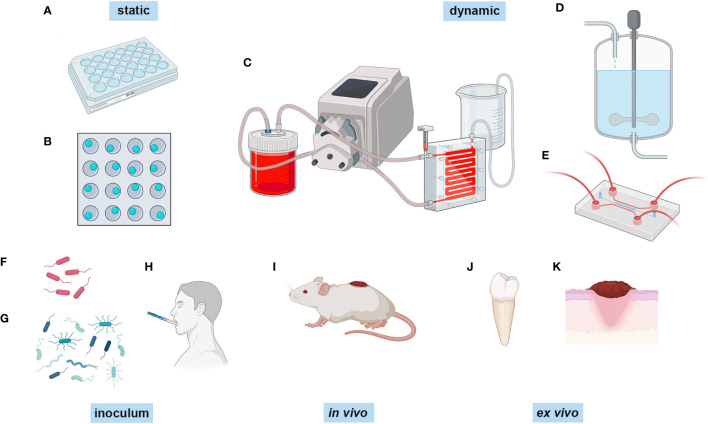
Different methods of biofilm growth. Biofilms can be grown in a number of different ways. Biofilms can be grown statically in **(A)** microtitre plates and **(B)** microtitre plates with glass beads, titanium discs or hydroxyapatite discs in the bottom of the well. They can be grown dynamically in **(C)** flow cells combined wuth peristaltic pumps, **(D)** in a constant depth film fermenter, and **(E)** in a microfluidic system such as the BioFlux. The inoculum can range from **(F)** single and **(G)** mixed species polymicrobial consortia, and inoculating collected samples from participants, such as **(H)** collected saliva. *In vivo* modelling is generally peformed on **(I)** live animals, and *ex vivo* modelling is performed on extracted samples such as **(J)** teeth and **(K)** skin samples, and also skin models. Created with BioRender.com.

The most simple biofilm experiment is the microtitre plate assay where biofilms are formed on the bottom of a microtitre plate (O’Toole, 2011) and are typically stained with crystal violet to assay biofilm biomass (**Figure 1A**). Additionally, different substrates can be added to the bottom of the well for biofilms to adhere to and grow on, such as glass beads, titanium discs and hydroxyapatite discs (**Figure 1B**), which can be removed for imaging or for other types of quantative analysis. Another simple model is using an 8 chamber glass slide, where biofilms can be grown and directly imaged (Jurcisek et al., 2011). Although these methods are quick, cheap, and easy, these are typically not considered to be true mature biofilms as they are statically grown sedimentation cultures and are not exposed to sheer forces that are typical at many *in vivo* sites (e.g., urinary tract, gut, oral cavity). Furthermore, static cultures also limit the availability of nutrients and build up of bioproducts, as the culture medium is generally only changed manually every 12-48 hours.

### 2.2 Dynamic growth

#### 2.2.1 Flow cells

A more advanced system for growing biofilms, that overcomes the limited nutrient availability and allows them to reach maturation, is the use of flow cell systems or constant flow chambers (Peters and Wimpenny, 1988) (**Figure 1C**). This is a method of growing and evaluating biofilms under hydrodynamic conditions. This comprises a basic set up with a flask for growth media which is fed to the biofilm that grows within a chamber by a peristaltic pump and syringe bubble trap, followed by a waste bottle (Nielsen et al., 2011; Crusz et al., 2012) . The growth medium can be replaced with the dye of choice for imaging, the flow cell can then be removed from the system and biofilm images can be captured directly using fluorescence and confocal microscopy (Pamp et al., 2009; Nielsen et al., 2011) . One benefit of the flow cell system is that the chambers and the equipment can be sterilised by autoclave, therefore facilitating multiple reuse and subsequently reducing laboratory costs. However, this is also one of the disadvantages as this can quickly introduce contamination to the system if inadequate sterilisation has been performed (Palmer, 1999). Nonetheless, flow cells are a satisfactory choice if the user wants to analyse biofilm growth in real-time.

#### 2.2.2 Bioreactor systems

Another method of dynamic growth of biofilms is by using systems such as the constant depth film fermentor (CDFF) (**Figure 1D**). These have been used for decades to grow biofilms, and consist of ports for nutrient and gas exchange, housing for substrates which are inserted to a known depth, and a turntable with a scraper which keeps the biofilm at a constant depth, if required (Pratten, 2007). Biofilms can be pulsed with nutrients or inhibitors (e.g. antimicrobials) as there are additional ports, and the substrates upon which the biofilms have formed can be removed for additional analysis. These types of biofilm model benefit from constant nutrient availability, and are well-suited for use in oral biofilm modelling as the constant flow mimics the saliva coating the tooth surface (Wilson, 1999; Hope and Wilson, 2006; Pratten, 2007; Sousa et al., 2022) and for wound biofilms which grow at the air-liquid interface and are constantly nourished from the exudate beneath (Duckworth et al., 2018). However, there are issues with heterogeneity of the biofilms that are formed due to either minor changes in the number of bacteria in a defined consortium inoculum, or the variability of human samples that are inoculated (Hope et al., 2012) which can affect the final biofilm composition.

#### 2.2.3 Microfluidic systems

In recent years, the state of the art in experimental biofilm modelling and analysis is through the use of microfluidics (**Figure 1E**), which includes devices such as Bioflux (Fluxion Biosciences) (Pouget et al., 2021) and BiofilmChip (Blanco-Cabra et al., 2021). Microfluidics is the use of small channels, microns in thickness, and a constant, low volume flow of fluid that facilitates small-scale experiments, not only for biofilm development but also for other applications such as organ-on-a-chip and cancer studies (Li et al., 2016). In the case of the Bioflux system, the set-up allows for growth at different temperatures using a heating plate, different flow rates dependent on dyne pressure, different air requirements by the addition of gas tanks, and the double inlet of the Bioflux allows for switching between growth media. Biofilms can be grown either by binding and forming within the glass chamber alone or by seeding the channels with monolayers of cells (Tremblay et al., 2015). The inlets can also be used to pulse biofilms with antimicrobials (Naudin et al., 2019; Zhang et al., 2019a; Czechowicz et al., 2022). The BiofilmChip is a similar concept to the Bioflux, except it does not have the same plate-type technology. However, it does allow for the analysis of biofilm growth using electrochemical impedance spectroscopy (EIS) technology (Blanco-Cabra et al., 2021), which is the same technology used in the closed-system microtitre plate biofilm growth dynamic analysis xCELLigence Real Time Cell Analyzer (RTCA) (Acea Biosciences) (van Duuren et al., 2017). EIS uses electrical currents applied to a sample and measures the response using sensors to detect biofilm growth at the solid-liquid interphase (McGlennen et al., 2023). This allows for dynamic growth analysis without having to use a confocal microscope, although this can still be utilised if required. Arguably, one of the major advantages of microfluidic devices is the ability to image the biofilm directly *in situ* in real-time (Brown et al., 2019; Straub et al., 2020; Corsini et al., 2021) using a number of different probes and stains at single-cell resolution using confocal microscopy to generate complex images of the biofilm structure and its components. Whilst one advantage is being able to use low inoculation and culture volumes, therefore accommodating small clinical samples, this also comes with small output volumes for downstream sampling. However, one study using a robotic sampler for non-destructive sampling of the biofilm has shown that very small 10 μl samples can be retrieved and used directly for 16S rRNA gene sequencing (Hansen et al., 2019), along with high performance liquid chromatography (HPLC). Microfluidic systems have also been integrated with downstream applications *in situ* by pairing real-time biofilm growth in a vacuum compatible microfluidic reactor and time-of-flight secondary ion mass spectrometry (ToF-SIMS) (Hua et al., 2015) to assess spatial chemical mapping within the biofilm.

One of the disadvantages of using these biofilm models is that the biofilms must be adhered to a surface, therefore not considering non-surface associated biofilms. However, this can be overcome by using droplet microfluidics, a method of using small, encapsulated droplets to grow bacteria in biofilms. Specialised platforms are required to carry out these experiments, therefore they may not currently be accessible to most laboratories. This method is in a phase of optimisation for biofilm development, with two studies using *Bacillus subtilis* and *P. aeruginosa* to develop biofilms in droplets (Chang et al., 2015; Jin et al., 2018). Both studies have shown that biofilm development is highly dependent on the size of the droplet, and that uniformity of droplet size to be consistent across the samples is one major limitation of this method of biofilm growth. However, this method is very high throughput, with the ability to assess thousands, even millions, of droplet biofilms at one time.

### 2.3 Single, mixed species and microcosm biofilm models

Traditionally, biofilm investigations focused on single species of bacteria and characterised their ability to form biofilms as a mono-species model (**Figure 1F**). This has been hugely beneficial, and continues to increase our understanding of bacterial biofilm development immensely. Growing biofilms as single species allows for the optimisation and assessment of different biofilm growth models and assessing antimicrobials (Sena et al., 2006; Wang et al., 2019), as variability is lower in single species, therefore a more reliable comparison can be made. However, it has been shown that growth as single-species is not the prefered way that bacteria thrive and survive in natural biofilms, such as in dental plaque (Periasamy and Kolenbrander, 2010). As mentioned previously, biofilms grow in a state of symbiosis, sharing nutrients and antimicrobial resistance, which makes them far more resilient to environmental stresses (Lee et al., 2014). The *in vitro* growth of multispecies biofilms is essentially an effort to replicate the environment in which the biofilms grow. Dual-species and multispecies microcosm biofilms (**Figure 1G**) have been used to investigate biofilms related to different infections or body locations, such as dual-species *S. aureus* and *Candida aureus* infection (Tran et al., 2022), a nine species supragingival model (Bradshaw et al., 1989), a four species catheter-associated infection model (Hou et al., 2022) , a dual species *E. coli* and *Enterococcus faecalis* gut biofilm model (Govindarajan et al., 2022). *In vitro* biofilm models can also be generated using samples collected from patients/participants as the inoculating fluid, as opposed to a defined consortium of bacteria. The most common is retrievied saliva (**Figure 1H**) and plaque from the oral cavity (Signori et al., 2016), but studies have also used vaginal fluid from patients with chronic candidiasis (Cordeiro et al., 2020).

### 2.4 Modelling host-microbe interactions

*In vitro* modelling of biofilm infections is limited in that it is purely a prediction of what will happen *in situ*, and does not take into account the host response to biofilm infection. *In vivo* animal models have been used to grow biofilms in a more realistic and translational manner, when compared to *in vitro* biofilm modelling. For wound biofilm modelling (reviewed in (Ganesh et al., 2015)) pigs, mice, rats and even drosophila were inflicted with a wound (**Figure 1I**) which was then challenged with differing combinations of bacteria that are common in wound infections (*P. aeruginosa, S. aureus*, and *Acinetobacter baumanii* for example). *In vivo* modelling has also been utilised to study prosthetic joint infections, such as in mouse models where the animals receive a prosthetic joint and are then given an intra-articular injection of bacteria (Carli et al., 2017), or using horse or pig synovial fluid to mimic non-surface attached biofilms in the joint space (Gilbertie et al., 2019) . Host-microbe interactions can be modelled *in vivo* in animal models, although this is falling out of favour because of the obvious moral and ethical issues associated with these types of experiments. *Ex vivo* modelling using mock tissue systems or collected human tissues as the substrate is also gaining traction as an acceptable replacement (Corzo-León et al., 2019).

Whilst extracted teeth have been used for decades to grow oral biofilms (**Figure 1J**), *ex vivo* biofilm modelling is advancing rapidly with the advent of new technologies. Simpler models include *P. aeruginosa* biofilms that have been successfully modelled using synthetic cystic fibrosis sputum medium (Davies et al., 2017; De Bleeckere et al., 2023). Most commonly, *ex vivo* biofilm experiments use explanted material such as explanted porcine skin (Yang et al., 2013; Wilkinson et al., 2018) to study wound biofilms (**Figure 1K**), explanted corneal samples from rabbits and humans to study biofilms that contribute to keratitis (Pinnock et al., 2017) , explanted rat bone to study osteomyelitis (Junka et al., 2017), explanted porcine bronchiolar tissue to study cystic fibrosis biofilms (Harrison and Diggle, 2016), and explanted porcine heart valves (Chuang-Smith et al., 2010). More recently, reconstructed human epidermis (RHE) from stem cells known as Labskin has been used to model host-microbe interactions (Larson et al., 2021), therefore overcoming the requirement to harvest live tissue from animals or humans.

## 3 Image analysis techniques

As with biofilm modelling, there are basic techniques which offer low-resolution imaging, graduating to more advanced techniques which offer high-resolution information on the overall biofilm structure and its components (**Table 1**). Light microscopy is the most basic biofilm imaging technique and is useful as a way of visually confirming if biofilm material is present on the substrate being analysed (Bakke and Olsson, 1986). It is cheap and easy to perform – almost all microbiology laboratories will have a light microscope. However, this microscopy method is limited in biofilm analysis, as the resolution is far too low to elucidate the complex structures within these models.

### 3.1 Fluorescence-based microscopy

A step-up from the light microscope is the use of fluorescence microscopes, coupled with fluorescence staining. A number of fluorophores can be used, such as SYTO-9 and propidium iodide for live/dead staining, or probes using DNA tags for specific species staining (i.e., fluorescence *in situ* hybridisation (FISH) staining). Fluorescence microscopy is often paired with higher-resolution microscopy as a way of identifying live and dead bacteria at the surface of the biofilm prior to electron microscopy (Hassan et al., 2010). Fluorescence microscopy has been assessed in comparison to confocal laser scanning microscopy (CLSM) in an *in vitro* CDFF biofilm model, and was shown to image to greater depths than CLSM without a loss of resolution in this biofilm type (Vroom et al., 1999) . Not only can fluorescence microscopy be used to assess *in vitro* biofilms, but also removed medical devices to look for residual biofilm contamination (Wong et al., 2020). One of the main limitations to fluorescence microscopy is the limited time that fluorescence remains active – fluorophores are easily bleached by excitation under the microscope, limiting the time to analyse. Additionally, fluorescence staining overlap is a limitation here, with some probes having overlapping excitation/emission wavelengths which can cause issues with acquiring and analysing images.

**Figure 2:**
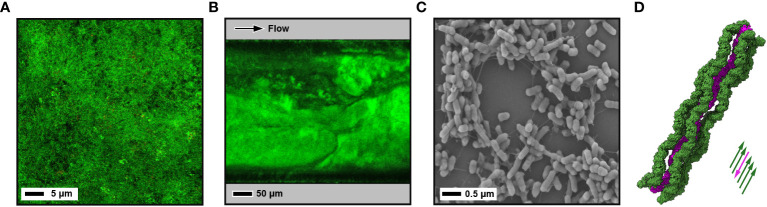
Images of biofilms using different techniques. **(A)** CLSM image of a 10-day old microcosm biofilm grown on a hydroxyapatite disc from a saliva sample inoculum. Biofilm is stained with LIVE/DEAD™ BacLight™ Bacterial Viability Kit (green - live; red - dead). **(B)** CLSM image of a mature 4-day old *E*. *coli* W strain biofilm grown under shear flow with a BioFlux microfluidic system. Bacterial membranes are stained with FilmTracer™ FM™ 1-43 dye. **(C)** Negative stain SEM image of a 1-day old enterotixogenic *E*. *coli* (ETEC) H10407 strain biofilm showing microcolony formation. **(D)** Cryo-EM derived atomic structure of bundled *in situ* archaeal bundling pili from *Pyrobaculum calidifontis* at 4 Å resolution (PDB ID: 7ueg) (Wang et al., 2022) . One extended pilus (purple) is surrounded by five other pili running in the opposite direction (green).

Fluorescence microscopy and CLSM share similar attributes, as fluorescent probes and stains can also be used in CLSM. Although CLSM uses fluorescence to visualise samples, it is different from fluorescence microscopy due to the pinpoint laser which improves resolution and the scanning of the laser through the sample on the *x, y* and *z* planes which provides *z*-stack images which can be reconstructed using open-source software such as COMSTAT2 (Vorregaard, 2008; Samarian et al., 2014) and BiofilmQ (Hartmann et al., 2021) . The information obtained from *z*-stack image analysis can provide information on biofilm depth, biomass, and surface area which is useful to compare biofilm characteristics when exposed to different molecules, along with live/dead staining (**Figures 2A, B**) which can show the distribution of bacterial viability within the biofilm (Bridier et al., 2010; Morris et al., 2020; Cleaver et al., 2021). Specific dyes and probes can be used, such as FM 1-43, fluorescent *in situ* hybridisation (FISH) probes (Thurnheer et al., 2004; Malic et al., 2009; Lebeer et al., 2011), and carboxy-SNARF (Corsini et al., 2021) for the monitoring of pH within the biofilm. However, CLSM suffers from photobleaching of samples, therefore the sampling of biofilms over a long period of time becomes a challenge. Lattice light sheet microscopy (LLSM) has been adapted to overcome this difficulty (Zhang et al., 2021a; Zhang et al., 2021b). LLSM has high resolution, both spatially and temporally, and it has low phototoxicity and photobleaching. Its benefits for biofilm experiments have been limited until recently, due to the water-immersion objectives on the microscope, however this microscopy methodology has been adapted to be used for biofilm dynamic analysis by pairing with hermetically sealed flow cells (Zhang et al., 2021b). An open-source analytical pipeline has also been developed to analyse 3D images captured using LLSM (Zhang et al., 2022). To achieve good quality images, samples must be grown on transparent media, or kept at a thin depth, which is a current limitation of this method.

### 3.2 Electron microscopy

The most advanced methods for microscopic analyses of biofilm samples are high-resolution scanning electron microscopy (SEM) and transmission electron microscopy (TEM). The main difference between the two methods is that in SEM, 3D surface images are produced through the detection of secondary electrons emitted from the sample surface after excitation from a primary electron beam (Garcez et al., 2013; Hung et al., 2013; Nishitani et al., 2015). On the otherhand, in TEM the primary beam passes through a sample, is diffracted, and then refocussed to produce an image as a 2D projection (Richardson et al., 2009; Sugimoto et al., 2016).

SEM is a well-established method that has been used extensively to assess the relationship between bacteria within a biofilm and with the substrate surface (**Figure 2C**). Conventional negative stain SEM has been expanded upon to include field emission SEM (FE-SEM), variable pressure SEM (VP-SEM) and cryo-SEM. Although negative stain SEM is relatively low in resolution (up to ~100 nm) it has high magnification (up to x30,000) (Relucenti et al., 2021), and the preparation and preservation of samples allows the intricate water channels and the biofilm matrix to be clearly visualised. However, preparative steps can lead to loss of the sample through fixation and drying, and the use of metal coating. Ionic coating of biofilms has overcome this to some extent (Asahi et al., 2015), but cryo-SEM is now considerd much more superior, involving freeze fracture of the sample which enhances the retention of the inner structures (Hrubanova et al., 2016; Hrubanova et al., 2018) (**Figure 2D**). Although SEM alone can only provide a surface image, focussed ion beam (FIB) milling can be used to remove ~10 nm thick sections from biofilms, and when coupled iteratively with SEM imaging (FIB-SEM), this can be used to investigate the subsurface structure and create 3D tomographical biofilm reconstructrions (Alhede et al., 2012). SEM is an incredibly useful tool in the investigation of biofilms and has been used in a range of different ways. For example, it has been used to characterise biofilm structure and formation (Fleeman et al., 2023), to assess the effects of antimicrobials on biofilms formed on different surfaces (Gomes and Mergulhão, 2017), and also clinically to assess the biofilm removal efficiency of different disruption techniques (Vyas et al., 2016) and for the detection of biofilms in ureteral stents in renal transplant patients (Barajas-García et al., 2023).

TEM has been used much less-frequently than SEM for the analysis of biofilms due to thin samples being required, and it has generally only been useful for imaging surface and internal structures of bacterial cells. However, TEM has extremely high spatial resolution (~1 Å) and with the emergence of cryo-TEM as a powerful tool for structural biology, cryo-TEM approaches have the potential to deliver *in situ* atomic resolution details within biofilms (Cossa and Trépout, 2022). Although not mature biofilms, *Pyrobaculum calidifontis* archaea, aggregated through intertwined intercellular pili, have been imaged by cryo-TEM, and using helical reconstruction the structure of these bundled pili has been modelled at 4 Å resolution (Wang et al., 2022). Likewise, using FIB milling with cryo-electron tomography (cryo-ET) to examine biofilm-like floccules of *P. aeruginosa* cells has shown how the surface protein CdrA localises at the intercellular interface and mediates bacterial aggregation (Melia et al., 2021). The ability to section mature biofilms in a non-disruptive fashion is a current draw back in being able to study their high resolution features *in situ*. However, by growing biofilms directly onto EM grids and/or using advanced sectioning approaches such as FIB milling, it may be possible in the future to overcome these barriers.

### 3.3 Atomic force microscopy

Another method for the microscopic analysis of biofilms is atomic force microscopy (AFM), which can produce an image by scanning a small cantilever over the surface of a sample, and measuring the force between the two (Hiesgen and Friedrich, 2011). This can be achieved through tapping mode where the cantilever is intermittently oscilated up and down, and contact mode where the cantilever is dragged across the sample and is in constant contact with the biofilm. Although resolution is generally low in the lateral plane (~2-30 nm), atomic resolution (~1 Å) can be achieved in the vertical direction. AFM has been used in biofilm analysis to study the growth of different bacteria on different substrates, namely investigating bacterial adhesion and morphology, the interaction of bacteria within polymicrobial biofilms, and the effect of surface treatments and antimicrobials (Beech et al., 1996; Chaw et al., 2005; Núñez et al., 2005; Ahimou et al., 2007; Oh et al., 2009; Germano et al., 2013). There are many advantages to using AFM in biofilm studies, including the ability to image nanoscale structures, simplified sample preparation, and its non-destructive nature (Vahabi et al., 2013). One disadvantage to AFM is that biofilms are often dried-out during the process, therefore often losing the integral structures and topography of biofilms, but one study has overcome this by creating a moist bioreactor that inhibits this drying process (Ahimou et al., 2007). Another disadvantage is that the sample must be well-adhered to the surface, which makes studying cell-cell interactions within non-adhered biofilms impossible with this technique.

### 3.4 Scanning electrochemical microscopy

Another method of imaging of biofilms is by using scanning electrochemical microscopy (SECM). This method of microscopy assesses bioelectric currents that are produced by bacteria within biofilms using small microelectrodes, similar to those used in EIS (Revsbech, 2005; Huang et al., 2018; Caniglia and Kranz, 2020) . The probe scans the biofilm surface and retrieves information regarding the redox processes around the sample and can provide a micron-scale 3D map of the environment (Darch and Koley, 2018). This methodology can be used to assess the growth and metabolic potential of biofilms (reviewed extensively by (Zhou et al., 2022)), but also to measure the antimicrobial effects of electrical stimulation on the biofilm (Del Pozo et al., 2009; Darvishi et al., 2021; Darvishi and Girault, 2023). SECM can be combined with other microscopic techniques, such as AFM (Huang et al., 2018) and CLSM (Cannan et al., 2002). One of the major advantages of SECM is that it is non-destructive and can be used in real-time to assess different molecules at the surface of the biofilm, such as virulence factors including pyocyanin in *P. aeruginosa* (Koley et al., 2011). There are some limitations to SECM, mainly the long scanning times, the inability to scan large surfaces and the inability to scan rough surfaces (Santana Santos et al., 2023), however the hybridisation of this method with others has the potential to overcome these limitations.

## 4 Genetic and molecular analysis techniques

### 4.1 Sequencing analysis

Molecular biology techniques range from simple targeted polymerase chain reactions (PCR) to advanced metatranscriptomic analysis (**Table 1**). All techniques have their place within the scope of the experiment being carried out, and some answer “what bacteria are there?” and others “what are those bacteria doing?”. All molecular biology investigations require a solid research hypothesis before being performed, as the vast amount of data derived, particularly from high throughput sequencing techniques, can be difficult to decipher without one. Their application in biofilm research is extensive, particularly in dental biofilm samples where 26% of the oral microbiome is unculturable (https://www.homd.org/; last accessed 20^th^ October 2023), therefore allowing the detection of bacteria that would normally be missed in conventional microbiology techniques (Chen et al., 2010; Dewhirst et al., 2010).

Sequencing of the variable 16S rRNA gene is a relatively simple method of identifying bacteria in biofilms down to the genus level confidently (Dempsey et al., 2007; Swearingen et al., 2016; Cleaver et al., 2019). However, 16S rRNA gene sequencing can be limited by several variables, such as the bias of primer pairs that are used for amplification (specifically those that are universal primers (Morales and Holben, 2009)), the variable region that is targeted (Johnson et al., 2019) , and also the curated database that is used to identify sequences to species level. A study by Johnson and colleagues (Johnson et al., 2019) also suggested that the most accurate method to discriminate down to species level is to sequence the entire 16S rRNA gene using long sequence reads, which can be produced by platforms such as Oxford Nanopore (Tianyuan et al., 2023). Molecular identification of fungal species within a biofilm requires sequencing of the nuclear ribosomal internal transcribed spacer (ITS) region (Nilsson et al., 2009), and cannot be performed concurrently with 16S rRNA gene sequencing.

To overcome these limitations, one might choose to use metagenomic next generation sequencing to sequence all the genes present within a polymicrobial biofilm sample. A study comparing the two showed that metagenomic sequencing has a much higher level of taxonomic diversity and specificity, and that 16S rRNA gene sequencing showed broader community composition and is less specific (Poretsky et al., 2014). Metagenomic sequencing also gives information on antimicrobial resistance within a biofilm sample, and also allows for predicting functionality of the biofilm using the Kyoto Encyclopedia of Genes and Genomes (KEGG) (Matsumoto et al., 2021; Yen and Johnson, 2021). Whole genome sequencing has its place in biofilm analysis, however, the presence of a gene within the genome does not necessarily mean that it is being transcribed.

### 4.2 Transcriptomics

Transcriptomics is the analysis of the complete mRNA transcripts of cells and provides information on differential gene expression of single cell types or polymicrobial communities and is an advancing method of identifying the functionality and activity of biofilms (**Table 1**). Sequencing the mRNA transcripts of cells allows both dual sequencing of bacterial and fungal biofilms, however, the database used to blast sequences must include both bacterial and fungal genes. The switch from planktonic to sessile growth is tightly regulated by bacterial gene expression, and this has been shown in different biofilm models (Oggioni et al., 2006; Shemesh et al., 2007; Sánchez et al., 2019) and biofilm growth dynamics are different from planktonic cell growth dynamics (Resch et al., 2005; Robertsson et al., 2021; Zheng et al., 2022). Metatranscriptomics is a powerful tool in microbiology research for the analysis of *in vitro* biofilm growth models (Zhang et al., 2019b), but also for examination of clinical samples, such as dental plaque (Edlund et al., 2018; Huang et al., 2021), prosthetic joint infections (Goswami et al., 2021), and host-microbiome interactions in diabetic wound biofilms (Malone et al., 2022).

**Figure 3:**
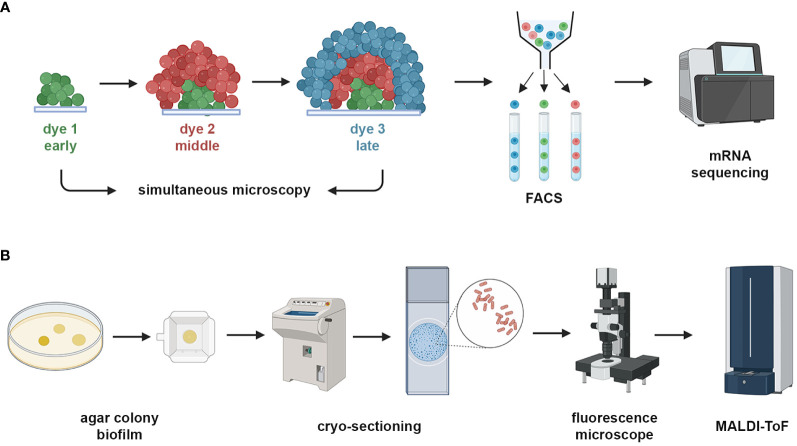
Spatial and functional analysis of biofilms. Imaging can be coupled with transcriptomics and mass spectrometry to assess the functionality of biofilm samples. **(A)** A schematic view of RAINBOW-seq, where different fluorescent dyes are sequentially added to a biofilm which is imaged at regular intervals, after which the biofilm is subjected to FACS cell sorting and then low quantity mRNA sequencing is performed which is mapped back to the image using bioinformatic gating parameters (Dar et al., 2021) . **(B)** A schematic view of imaging mass spectroscopy, where agar grown colony biofilms that are eGFP fluorescence tagged are grown and cryosectioned into thin slices, which are imaged and then subjected to MALDI-ToF mass spectrometry with post-processing mapping to the image. Created with BioRender.com.

One of the limitations of biofilm metatranscriptomics has been the inability to resolve spatial functionality within the biofilm structure. However, spatial transcriptomics is now a fast-developing method which is being used to understand how microbes behave in different topographical locations within the biofilm (Dar et al., 2021a). A range of different methodologies to achieve this have been reported in the literature. One group have developed RAINBOW-seq, a method where fluorescently labelled dyes are used to label bacteria spatially within a biofilm (surface, middle, interior) during growth within a microfluidic system (**Figure 3A**). These are then sorted by fluorescence-activated cell sorting (FACS), after which RNA-seq is performed (Wang et al., 2023). Another group has grown biofilms in a drip-flow chamber on stainless steel coupons, which were then cryo-sectioned and slices from the surface, middle and interior of the biofilm and then analysed with microarrays (Heacock-Kang et al., 2017). Sequencing of RNA transcripts from colony biofilms has also been carried out, whereby filter membranes seeded with *E. coli* were grown on solid agar plates at regular intervals from 12-72 hours and the colonies at these time periods were then subject to transcriptomics, adaptive microscopy, metabolomics using ultra-high performance liquid chromatography (UPLC), and oxygen measurements (Díaz-Pascual et al., 2021). Although technically not a true biofilm, this methodology gives a good picture of colony biofilm growth and functionality over time. Agar block biofilm assays (ABBA) have also been used to grow *P. aeruginosa* biofilms in chambered coverslips, which were then hybridised with fluorescent probes targeted to genes of interest and imaged using CLSM (Livingston et al., 2022) to visualise gene expression within the biofilms. Furthermore, a set of 105 unique genes, representing physiology and virulence, from *P. aeruginosa* biofilms grown in coverslip chambers have been spatially imaged using par-seqFISH, which is a new method of parallel sequential fluorescence *in situ* hybridisation CLSM (Dar et al., 2021).

However, these tehniques so far can only resolve regions within biofilms rather than at the single cell level. The development of single cell transcriptomics from bacterial cells has been challenging, as bacterial cells contain low levels of mRNA, the mRNA does not have a poly(A) tail making some sequencing techniques unsuitable, and bacterial mRNA is notoriously unstable (Homberger et al., 2022). Overcoming these challenges will allow significant advancements in our insight into bacterial functionality within these complex biofilm communities.

### 4.3 Proteomics and metabolomics

Sequencing bacterial mRNA from biofilm samples can give information on which genes are being expressed but does not necessarily equate to translation of those genes and how the molecules that are translated are utilised, especially in polymicrobial biofilm samples (**Table 1**). However, analysing the proteome and metabolome of biofilms, with or without gene expression data, is able to provide a much more complete picture of the biofilm environment.

Bacterial proteomics is the analysis of all the proteins that are present in a sample and can be used to analyse the degradation of nutrients by bacteria. Proteomic analysis of biofilms has been used to identify key proteins that are essential in complex biofilm networks, including; in the EPS (Egorova et al., 2022) , required for temperature adaptation stress (Lee and Wang, 2020) , required for adaptation to antimicrobial treatment (MaChado and Coquet, 2016) , and that change with biofilm development (Serra et al., 2008; Suriyanarayanan et al., 2018) and aging (Rahman et al., 2022), and between planktonic and biofilm conditions (Khan et al., 2019; Suryaletha et al., 2019; Llama-Palacios et al., 2020; Tang et al., 2020). These studies use a range of different mass spectrometry methodologies, including tandem liquid chromatography (LC-MS/MS) alone (Lee and Wang, 2020) or coupled with nano-high performance liquid chromatography (HPLC) (Egorova et al., 2022), lab-on-a-chip and XCT mass spectrometry (MaChado and Coquet, 2016), tandem mass tag (TMT) mass spectrometry (Rahman et al., 2022), fourier transform infrared spectroscopy (FT-IR) (Serra et al., 2008), nano-LC/MS coupled with quadrupole-ToF (Q-ToF) (Suryaletha et al., 2019) , iTRAQ labelling plus LC-MS/MS for quantitative proteomics, and matrix-assisted laser desorption ionisation time-of-flight (MALDI-ToF) mass spectrometry (Llama-Palacios et al., 2020; Tang et al., 2020). MALDI-ToF analysis of bacteria is routinely used in clinical diagnostic laboratories to identify bacterial species, and this method is increasingly being developed to discriminate between isolates and their status as biofilm-producers or non-producers (Caputo et al., 2018), which will aid in establishing effective antimicrobial therapy.

Proteomics has also been coupled with imaging analysis to investigate the proteome spatially within the biofilm. One of the most utilised methods of achieving this is with MALDI-ToF imaging mass spectrometry (MALDI-ToF IMS) (Floyd et al., 2015; Rivera et al., 2022), whereby biofilms are cryo-sectioned and imaged with a fluorescent microscope prior to MALDI-ToF (Rivera et al., 2022) (**Figure 3B**) or biofilms are grown on slides and subjected to electron microscopy and MALDI-ToF (Floyd et al., 2015). Another method is laser ablation sample transfer (LAST), where biofilms can be grown on transwell membranes which are then laid on a microscope slide on the anoxic or oxic side and subjected to LC-MS/MS (Pulukkody et al., 2021). The latter method does not employ image analysis but rather proteomic analysis based on oxygen availability within the biofilm.

Bacterial metabolomics is the identification and quantification of metabolites within a sample that have either been produced by bacteria (metabolised) or are a result of the breakdown of a substrate into other downstream products (catabolised) by bacteria. This can be; (1) targeted, where the absolute concentration of a metabolite is quantified using labelled isotopes, or (2) untargeted, where metabolites are semi-quantitatively quantified using a control sample. Mass spectrometry is used regularly to undertake metabolomic analysis of biofilm samples, however nuclear magnetic resonance (NMR) spectroscopy has been used more frequently in recent biofilm metabolomic studies (Renslow et al., 2013; Cleaver et al., 2019; Cleaver et al., 2021; Czajkowska et al., 2021; Leggett et al., 2022). Whereas mass spectrometry assesses the mass to charge ratio of charged molecules to determine molecular identity (Emonet et al., 2010) , NMR detects changes in the local electronic environment and can provide fingerprint spectra for specific molecular structures (Singh and Singh, 2022). Although NMR is more reproducible than mass spectrometry, does not destroy the sample, and is more efficient to run, one of its limitations is that it is less sensitive, where mass spectrometry approaches tend to identify a higher range of metabolites (Emwas et al., 2013).

## 5 Conclusions

Biofilms are incredibly complex and dynamic structures, and cause a range of difficult to treat medical infections. Advancements in biofilm research is fast-paced and new ways to study biofilms are in constant development. This review has detailed the numerous ways to grow and image biofilms, and investigate the multifaceted dynamics of biofilm behaviour. It is important to use a technique that is appropriate for the biofilm infection being studied and the methods that are chosen ultimately depend on the hypotheses and questions that are aiming to be answered, and these should be carefully considered.

## Author contributions

LC: Conceptualization, Writing – original draft, Writing – review & editing. JG: Conceptualization, Funding acquisition, Writing – original draft, Writing – review & editing.
